# Ameliorative Effects of *Nypa fruticans* Leaf Extract on Anxiety and Depression: Evidence From In Vivo and In Silico Studies

**DOI:** 10.1155/bmri/1243521

**Published:** 2026-04-29

**Authors:** Farhana Islam, Sabbir Ahmed, Jannatul Ferdous, Mostafa Kamal, Asma Aktar, Fariya Islam Rodru, Md. Nurul Islam, Al Riyad Hasan

**Affiliations:** ^1^ Department of Pharmacy, Faculty of Biological Science and Technology, Jashore University of Science and Technology, Jashore, Bangladesh, just.edu.bd; ^2^ Department of Pharmacy, Mawlana Bhashani Science and Technology University, Tangail, Bangladesh, mbstu.ac.bd; ^3^ Department of Pharmacy, Jahangirnagar University, Savar, Dhaka, Bangladesh, juniv.edu; ^4^ Department of Footwear Technology, University of Dhaka, Dhaka, Bangladesh, du.ac.bd; ^5^ Department of Agriculture, Bangabandhu Sheikh Mujibur Rahman Agricultural University, Gazipur, Bangladesh, bsmrau.edu.bd

**Keywords:** antidepressant, anxiolytic, MD simulations, molecular docking, *Nypa fruticans*, sedative

## Abstract

**Ethnopharmacological Relevance:**

*Nypa fruticans* (Arecaceae) has long been used in traditional medicine for the management of anxiety, insomnia, and depressive symptoms. However, scientific validation of its neuropharmacological activities and active constituents is limited.

**Aim of the Study:**

This study evaluated the anxiolytic, antidepressant, and sedative activities of the ethyl acetate fraction of *Nypa fruticans* (NF) leaves (EaNFL) through in vivo behavioral models and in silico molecular docking (MD) and molecular dynamics simulations (MDS) to provide scientific support for its traditional CNS use.

**Materials and Methods:**

Neuropharmacological activities of EaNFL were assessed in mice using elevated plus maze (EPM), hole board test (HBT), open field test (OFT), hole cross test (HCT), tail suspension test (TST), and forced swimming test (FST). Major phytochemicals identified from EaNFL were subjected to MD analysis against voltage‐gated potassium channels (PDB: 4UUJ), GABA‐A receptors (PDB: 6X3X), and human serotonin transporter (PDB: 5I6X). Top‐binding compounds were further analyzed using MDS to assess receptor–ligand stability.

**Results:**

EaNFL (400 mg/kg) showed significant anxiolytic activity in EPM and HBT, dose‐dependent sedative properties in HCT and OFT, and notable antidepressant‐like effects in TST and FST. However, reduced locomotor activity may have contributed to some of the observed behavioral outcomes. MD revealed that myricetin (CID 5281672) and quercetin (CID 5280343) predicted stable interactions and favorable binding affinities with major CNS‐related receptors, as suggested by MDS analysis. Additionally, (−)‐epicatechin (CID 72276) and rosmarinic acid (CID 5281792) exhibited favorable receptor interactions, suggesting therapeutic potential in anxiety, insomnia, and depression.

**Conclusions:**

EaNFL possesses notable anxiolytic, sedative, and antidepressant‐like effects, supporting its traditional use in CNS disorders. MD and MDS findings suggest that myricetin, quercetin, and (−)‐epicatechin acid may be key bioactive constituents underlying these effects. Further studies are warranted to elucidate molecular mechanisms and explore its pharmaceutical potential.

## 1. Introduction

Anxiety and depression are among the most common mental health disorders globally, exerting a profound impact on both individuals and society. These conditions can lead to substantial impairments in quality of life, daily functioning, and emotional well‐being [1]. Understanding their nature and global burden is essential for the development of effective interventions and the improvement of mental health outcomes [2]. Anxiety and depressive disorders are highly prevalent, affecting an estimated 264 million and 300 million people worldwide, respectively, often co‐occurring and contributing significantly to global disability and disease burden [3–7].

Most of the available drug therapies for depression and anxiety are based on enhancing the short‐term levels of neurotransmitters in the brain, primarily utilizing the action of monoamine oxidase inhibitors (MAOIs), selective serotonin reuptake inhibitors (SSRIs), and serotonin (5‐HT), noradrenaline (NE) reuptake inhibitors (SNRIs), and GABA_A receptor agonist [8]. Conventional antidepressants like SSRIs and tricyclic antidepressants (TCAs) increase serotonin levels by blocking its reuptake [9]. GABA_A receptors are the main target of benzodiazepines’ enhancement of GABAergic transmission, which increases inhibitory neurotransmission and decreases anxiety [10]. SSRIs, TCAs, and benzodiazepines are frequently prescribed for the management of depression and anxiety; however, these treatments often exhibit limitations, including delayed therapeutic onset, detrimental side effects, and high rates of relapse after discontinuation [11–13].

Several drugs are available to treat anxiety and depression, but full and long‐term resolutions are not reached [3]. Currently, the initial therapy for depression and anxiety is SSRIs [14]. But such medications frequently lead to unwanted side effects, including dizziness, dry mouth, constipation, sexual dysfunction, weight gain, and excessive sweating [15]. Many adverse effects of benzodiazepines include mood disorders, sleepiness, shallow breathing, and cognitive impairments [16]. Significant adverse effects of all conventional antidepressants are also associated with weight gain, headaches, agitation, exhaustion, drowsiness, sedation, nausea and vomiting, and sexual dysfunction, which are responsible for poor patient compliance [17]. The shortcomings of currently available pharmaceutical treatments underscore the need for new, safer, and more potent therapeutic agents that are not only effective but also have a rapid onset of action and fewer adverse effects. So, there is a critical need for novel antidepressant medication with few adverse effects [3]. Medicinal plants’ wide phytochemical variety and long history of ethnomedical use have made them a useful source for drug discovery [18]. Research is presently focused on plant‐based medications because they are commonly obtained organically, have fewer adverse effects, are cheaper, and are safer when compared with synthetic drugs. Natural bioactive compounds can be assessed as safer and more effective therapeutic agents than synthetic ones, as they offer diverse potencies and are evaluated for better utilization [19].

Conventional medications for anxiety and depression often cause adverse effects such as sedation and dependence [17]. In contrast, in general, plant‐derived compounds are considered relatively safe, although toxicity may be dose‐ and compound‐dependent, and possess favorable pharmacokinetic properties [19]. Numerous plant‐based bioactive compounds, particularly flavonoids, terpenes, steroids, saponins, sugars, and alkaloids, exhibit anxiolytic and antidepressant effects through interactions with various receptors, suggesting their potential as natural alternatives to conventional antidepressants and anxiolytics [20]. These phytochemicals exert their effects by modulating neurotransmitter systems, including gamma‐aminobutyric acid (GABA) and serotonin pathways. Various screening models such as the tail suspension test (TST), forced swim test (FST), monoamine oxidase inhibition assay, open field test (OFT), and hole board test (HBT) are commonly employed to evaluate these activities [20, 21].

Moreover, many plant‐derived compounds exhibit structural resemblance to monoamines, which allows them to modulate neurotransmitter function and neurotrophic pathways positioning them as promising candidates for treating neuropsychiatric conditions [22]. Research has shown that deficiencies in monoamine neurotransmitters such as serotonin, dopamine, adrenaline, and NE are closely linked to anxiety, depression, and sleep disturbances [23].

*Nypa fruticans*(NF) Wurmb. (family Arecaceae), commonly known as the nipa palm, is widely used in traditional medicine in southern Bangladesh. Indigenous healers employ various parts of this mangrove palm to treat diverse ailments. The plant is rich in bioactive compounds such as flavonoids, polyphenols, tannins, terpenoids, steroids, alkaloids, cyanogenic glycosides, and saponins, which contribute to its broad pharmacological properties, including antioxidant, antidiabetic, antimicrobial, anticancer, anti‐inflammatory, and analgesic effects. Traditionally, *NF* has been used to alleviate conditions like stomach pain, diabetes, fever, toothache, hyperuricemia, and headaches [24–26]. However, treatment with the dried flower stalk of *NF* leaves significantly reduced NF‐*κ*B and iNOS expression, supporting its neuroprotective potential through TRPV1 modulation in peripheral nerve injury [27].

Given its diverse phytochemical composition and traditional medicinal uses, *NF* holds promise as a source of therapeutic agents for neuropsychiatric disorders, particularly depression and anxiety. However, to date, no scientific study has specifically evaluated its antidepressant and anxiolytic potential. This study is to evaluate the antidepressant, anxiolytic, and locomotor effects of the ethyl acetate leaf extract of *NF* (EaNFL) by *in vivo* and *in silico* methodologies. The objective is to identify the anxiolytic/antidepressant mechanisms of the bioactive compounds responsible for neuroprotection through computational analyses.

In addition to behavioral testing, Molecular docking (MD) was employed to predict the interactions between *NF* phytoconstituents and key neurological receptors. The docking targets included the human serotonin transporter (SERT), a primary target of SSRIs; voltage‐gated potassium ion channels, which regulate neuronal excitability; and gamma‐aminobutyric acid type A (GABA_A_) receptors, which mediate sedative and anxiolytic effects. Molecular dynamic simulations (MDS) were further carried out to examine how stably the identified compounds remained bound within the receptor complexes [28–30].

The combined use of in vivo and in silico methodologies offers a comprehensive strategy for investigating the neuropharmacological effects of EaNFL. By integrating behavioral outcomes with computational predictions of receptor interactions with molecular compound, this research aims to provide robust evidence for the potential of this plant as a candidate for developing alternative treatments for depression and anxiety. Such findings could pave the way for further pharmacological investigations and contribute to the ongoing search for plant‐based neurotherapeutics.

## 2. Materials and Methods

### 2.1. Chemicals and Reagents

Ethyl acetate was acquired from Sigma Chemical Company (St. Louis, MO, United States), while other chemicals were sourced from Merck (Darmstadt, Germany). Tween 80 was acquired from BDH Chemicals (Leicestershire, United Kingdom), and standard pharmaceuticals diazepam and imipramine hydrochloride were procured from Square Pharmaceuticals Ltd., Dhaka, Bangladesh. All reagents used were of analytical quality and conformed with standard specifications.

### 2.2. Collection, Identification, and Preparation of *NF* leaf extract

Green leaves of *NF* were collected in February 2024 from healthy plants in the Sundarbans, Khulna, Bangladesh, and authenticated by Khandakar Kamrul Islam, Senior Scientific Officer, Bangladesh National Herbarium (Voucher No. DACB: 92809). Ethyl acetate extract was prepared via cold maceration, followed by filtration, concentration, and storage for further analysis, as described in previous studies [31]. The collected leaves were cleaned and dried in the shade for 3 weeks and then milled into a fine powder with a laboratory grinding mill (Model 2000 LAB Eriez) and sieved on a 40‐mesh screen. The extraction process involved soaking 300 g of crushed *N. fruticans* in 1700 mL of ethyl acetate and leaving it macerate for 14 days while being shaken and stir occasionally. After that, the mixture was filtered onto Whatman Grade 1 filter paper (Sigma‐Aldrich, St. Louis, MO, United States). The filtrate was evaporated at 36°C and 40 rpm through a rotating vacuum evaporator (Buchi Rotavapor Model R‐124), and then it was dried in desiccators; 5.8 g of extract, yielding a 1.93% (w/w), was produced by this procedure and stored in an airtight container at 4°C [31].

### 2.3. Experimental animal

Swiss albino male mice (6–7 weeks old, 25–28 g) were obtained from the Animal Research Laboratory, Department of Pharmacy, Jahangirnagar University, Savar, Dhaka. The animals were housed in standard cages under controlled conditions (27^°^C ± 1^°^C, 55%–65% humidity, 12 h light/dark cycle) with ad libitum access to distilled water and a nutritionally balanced diet. All experimental procedures followed OECD guidelines Guideline No. 425 (Acute Oral Toxicity: Up‐and‐Down Procedure) and the European Community Directive (88/609/EEC, 1986), with ethical approval granted by the Ethical Review Committee of the Faculty of Biological Science and Technology, Jashore University of Science and Technology (Ref: ERC/FBST/JUST/2022–127).

### 2.4. Acute toxicity study

An acute toxicity study was conducted in our earlier research. Thirty‐five overnight‐fasted mice (25–30 g) of both sexes were randomly assigned to seven groups (*n* = 5). The extract of *N. fruticans* (EaNFL) was administered orally at doses of 200, 400, 1000, 2000, 3000, and 4000 mg/kg, with distilled water used as the vehicle. Animals were observed for 14 days for signs of toxicity, including changes in behavior, body weight, skin condition, lethargy, diarrhea, salivation, and mortality [31].

### 2.5. Animals and experimental design

In this study, mice were randomly assigned to experimental groups using a computer‐generated randomization schedule. All behavioral experiments were conducted by an experimenter blinded to the treatment groups. Animals were acclimatized to the testing room for at least 30 min prior to each experiment. For each experimental test, four groups were included: a control, a standard, and two test groups. The control group received distilled water orally at 10 mL/kg body weight (b.w.), while the test groups were administered EaNFL orally at doses of 200 and 400 mg/kg b.w. Based on the extract’s safety up to ~4000 mg/kg, doses of 200 and 400 mg/kg (≈1/20 and 1/10 of the maximum safe dose) were selected as low and moderate subtoxic levels. Diazepam (1 mg/kg, i.p.) served as the standard drug for the elevated plus maze (EPM), HBT, OFT, and hole cross test (HCT), whereas imipramine hydrochloride (10 mg/kg, i.p.) was used as the standard in the TST and FST. However, to avoid potential carry‐over effects, animals were not reused across behavioral experiments. Each behavioral test was performed using independent groups of fresh mice.

### 2.6. Antidepressant activity study

#### 2.6.1. TST

TST is a commonly employed and reliable technique to evaluate antidepressant effects, as outlined in the methodology of Ghosh et al. [32]. The tails of each mouse were affixed using adhesive tape and suspended at an elevation of 40 cm above the ground. The experimental procedure was initiated 30 min prior to the administration of all treatments. Within the 6‐min observation period, the first 2 min were considered the adjusting period and the last 4 min were recorded. Passively hanging mice were considered immobile.

#### 2.6.2. FST

FST was used to determine EaNFL’s antidepressant potential in mice. To familiarize the animals with the testing environment, an initial study was performed 1 day before the main experiment. The method described by Alves et al. [33] was utilized in this experiment. The experimental swimming apparatus was comprised of a glass tank (25 × 15 × 25 cm), filled with water up to 15 cm and kept at 25^°^C ± 2^°^C. After a 30‐min lapse post‐administration of the respective substances, each mouse was introduced into the tank for a period of 6 min. The initial 2 min were designated as an adjustment phase, while the subsequent 4 min were recorded as the duration of immobility.

### 2.7. Anxiolytic activity study

#### 2.7.1. EPM Test

The anxiolytic activity of EaNFL was evaluated using the EPM following the method of Sarkar et al. [34]. The apparatus consisted of two open arms (50 × 10 × 70 cm) and two closed arms (50 × 10 × 40 cm) arranged opposite each other with a central platform. The maze was elevated 50 cm above the floor and the experiments were conducted in a quiet, isolated room under dim lighting conditions. Mice were randomly assigned to groups (*n* = 6 per group) and treated with vehicle (control), EaNFL (200 and 400 mg/kg, p.o.), or diazepam (1 mg/kg, i.p.). Oral treatments were administered 60 min and intraperitoneal treatment 30 min prior to testing. Each mouse was placed individually on the central platform facing a closed arm and allowed to explore freely for 5 min. An arm entry was defined as the placement of all four paws into an arm. During the observation period, the time spent in the open arms and the number of open‐arm entries were recorded by an observer blinded to the treatment groups.

#### 2.7.2. HBT

The HBT was performed to evaluate exploratory behavior associated with anxiety following the method of Nobee et al. [35] with minor modification. The apparatus consisted of a wooden board (40 × 40 *c* *m*) elevated 25 cm above the floor, containing 16 evenly spaced holes (3 cm in diameter). The test was conducted in a quiet room under controlled lighting conditions (~120 lx). Mice were randomly assigned to treatment groups as described for the EPM test and received vehicle (control), EaNFL (200 and 400 mg/kg, p.o.), or diazepam (1 mg/kg, i.p.). Oral treatments were administered 60 min and intraperitoneal treatment 30 min prior to testing. Each mouse was placed individually at the center of the board and allowed to explore freely for 5 min. The number of head‐dipping behaviors (insertion of the head into a hole) was recorded by an observer blinded to the treatment groups.

### 2.8. Locomotor activity study

#### 2.8.1. OFT

OFT was conducted following the method described by Leaves et al. [36]. The open field apparatus employed in this investigation was constructed from plywood and comprised a white cubic enclosure of 60 cm in length, width, and height. The box was partitioned into 25 squares of identical dimensions, with each square measuring (12 × 12 cm). This device was used to evaluate both locomotor and emotional spontaneous behavior in mice. The open field surface was further separated into black and white square units. The experimental procedure took place in a quiet room with controlled lighting conditions. In the OFT, mice were positioned in the center, and their movement over the square blocks were observed. The number of blocks they traversed during the experiment was recorded. The recordings were then analyzed for a duration of 3 min at 0, 30, 60, 90, and 120‐min intervals [37].

#### 2.8.2. HCT

This experiment was conducted according to the method described by Apu et al. [38]. In this study, the cross cage used was made of stainless steel and had specific dimensions (30 × 20 × 14 cm). The cage was split into two chambers by a partition, which covered a hole (diameter: 3 cm, height: 7.5 cm). For the experiment, each mouse was placed at the midpoint of one side of the hole‐cross apparatus. Locomotor activity was assessed by counting the number of holes crossed as the animals moved between the two chambers. These observations were then analyzed for a duration of 3 min at 0, 30, 60, 90, and 120‐min intervals [39].

### 2.9. Statistical analysis

Statistical analyses were performed using IBM SPSS Statistics (version 23). Data are expressed as mean ± standard error of the mean (SEM). For comparisons among groups, one‐way analysis of variance (ANOVA) followed by Dunnett’s post hoc test was used. Locomotor activity data obtained from the OFT and HCT were recorded at multiple time points using the same animals; therefore, these data were analyzed using repeated‐measures ANOVA to account for within‐subject correlations across time. When significant effects were observed, Dunnett’s post hoc test was applied for multiple comparisons between treatment groups and the control group. This statistical approach ensured an appropriate evaluation of time‐dependent changes in locomotor activity and accurately reflected the repeated‐measures experimental design. Graphical representations were prepared using GraphPad Prism (version 9). Statistical significance relative to the control group was indicated as  ^∗^ *p* < 0.05,  ^∗∗^ *p* < 0.01,  ^∗∗∗^ *p* < 0.001.

### 2.10. In Silico studies

#### 2.10.1. Phytochemicals identified from EaNFL via HPLC and GC‐MS analysis

Phytochemicals identified via GC‐MS and HPLC, as detailed in our previous studies, were further subjected to MD and MDS [31].

#### 2.10.2. ADMET profiling

Phytochemicals from the EaNFL were evaluated for drug‐likeness and safety by comparing them with standard compounds. Pharmacokinetic properties were predicted using SwissADME and pkCSM [40, 41] while toxicity was assessed via the ProTox‐II server [42]. Compounds with high GI absorption, non‐toxicity, and compliance with Lipinski’s Rule of Five were selected for MD studies. Docking assessed their binding affinities to target proteins, helping identify promising candidates. This integrative analysis highlighted compounds with potential for further therapeutic development.

#### 2.10.3. Protein preparation

The three‐dimensional (3D) crystal structures of the SERT (PDB ID: 5I6X), potassium channel receptor (PDB ID: 4UUJ), and an additional target protein GABA A receptor (PDB ID: 6X3X) were retrieved from the Protein Data Bank (https://www.rcsb.org/). Initial structural preparation was conducted using the Protein Preparation Wizard from Maestro (Schrödinger Release 2021.4), which included the assignment of bond orders, the addition of missing hydrogen atoms, optimization of disulfide bonds, and correction of any incomplete side chains or loops via the Prime module. Water molecules beyond 5 Å from any hetero groups were removed, and any structurally irrelevant cofactors, metal ions, or non‐interacting ligands were excluded to avoid interference during docking. Additional refinement was carried out using BIOVIA Discovery Studio Visualizer (Version 2024) to eliminate non‐bonded residues and correct structural artifacts. The proteins were further subjected to energy minimization using Swiss‐PDB Viewer (v4.10), applying steepest descent methods and constrained RMSD thresholding to optimize the overall geometry. Finally, hydrogen bonding patterns were analyzed and assigned using PROPKA at pH 7.0, and the system was relaxed using the OPLS4 force field to prepare the proteins for MDS within the PyRx platform [43].

#### 2.10.4. Ligand preparation

3D structures of 10 bioactive compounds, as well as the standard drugs diazepam and imipramine, were obtained from the PubChem database (http://www.pubchem.ncbi.nlm.nih.gov/) in 3D‐SDF file format. Using PyRx software, the ligands were refined by generating appropriate ionization states and 3D conformers to ensure compatibility and accuracy in the subsequent docking studies [43].

#### 2.10.5. Active Site prediction and Receptor Grid generation

MD was conducted to evaluate the binding interactions between the selected ligands and the target proteins. The MD was performed using PyRx software, with grid boxes defined around the presumed active sites of each protein to ensure optimal ligand accommodation [44]. The grid box parameters were set as follows:

4UUJ: Center coordinates: x = 27.7202 Å, y = −19.8363 Å, z = −9.1975 Å; Grid box dimensions : x = 74.0020 Å, y = 64.6102 Å, z = 116.3451 Å.

5I6X: Center coordinates: x = −35.5063 Å, y = −15.1469 Å, z = 24.5528 Å; Grid box dimensions : x = 77.6251 Å, y = 70.0892 Å, z = 104.4510 Å.

6X3X: Center coordinates : x = 112.5933 Å, y = 113.5473 Å, z = 121.2039 Å; Grid box dimensions : x = 139.3626 Å, y = 138.4355 Å, z = 86.5109 Å.

These parameters ensured comprehensive coverage of the binding sites and accurate evaluation of ligand–protein interactions.

#### 2.10.6. Molecular Docking (MD)

MD was performed using PyRx (v0.9.8), which integrates AutoDock and AutoDock Vina for virtual screening. Ligands were energy‐minimized using the Universal Force Field (UFF) to ensure optimal geometry. The active site was defined by setting a grid box around the binding pocket based on literature or ligand‐bound coordinates. Docking simulations generated multiple binding poses, which were ranked by binding affinity (kcal/mol). The top‐scoring conformations were selected for visualization and interaction analysis [45].

#### 2.10.7. Molecular Dynamics Simulation (MDS)

A 100‐ns MDS was performed to evaluate the dynamic behavior and binding equilibrium of the protein–ligand complexes using the Desmond module of the Schrödinger Suite 2022.4 under an Ubuntu 22.04 operating environment. The protein–ligand complexes obtained from MD were used as the initial input structures for MDS. Ligand protonation states were assigned under physiological conditions using the Epik ionization engine implemented in Schrödinger LigPrep, generating possible ionization states at pH 7.0 ± 2.0 prior to docking. For polyphenolic compounds, hydroxyl groups were retained in their predominant neutral phenolic form within this physiological pH range. These protonation states were consistently maintained during both MD and subsequent MDs Each complex was solvated using the Simple Point‐Charge (SPC) water model, and an orthorhombic periodic boundary box with a 10 Å buffer distance from the solute was applied. Na^+^ and Cl^−^ ions were added to neutralize the system and maintain a physiological salt concentration of 0.15 M. The systems were minimized and equilibrated using the OPLS4 force field [46]. Simulations were carried out under an NPT ensemble, maintaining a temperature of 310 K and a pressure of 1.01325 bar [47]. Trajectory data were collected for subsequent structural and interaction analyses.

#### 2.10.8. Simulation Trajectory analysis

Schrodinger’s maestro interface version 9.5 was utilized to render all MDS snapshots. A simulation interaction diagram (SID) was set to calculate the simulation event generated by the MDS in the Schrödinger package. Using the trajectory output, the RMSD, RMSF, Rg, H‐ bond interaction, SASA, and PSA have been calculated.

## 3. Results

### 3.1. HPLC and GS‐MS–Based Phytochemicals identification

In our previously published study, a total of 23 phytochemicals were identified in EaNFL through HPLC and GC‐MS analyses [31]. A list of 23 phytochemicals are given in Table 1.

**Table 1:** Identified phytochemicals from EaNFL by GC‐MS and HPLC.

SN.	Chemical name
1.	(‐) Epicatechin (CID 72276)
2.	Caffeic acid (CID 689043)
3.	Rutin hydrate (CID 5280805)
4.	Rosmarinic acid (CID 5281792)
5.	Myricetin (CID 5281672)
6.	Kaempferol (CID 5280863)
7.	Quercetin (CID 5280343)
8.	trans‐Cinnamic acid (CID 444539)
9.	Guaiol (CID 227829)
10.	(‐)‐Aristolene (CID 530421)
11.	2,3‐dehydro‐4‐oxo‐.beta.‐ionone (CID 5363867)
12.	Neophytadiene (CID 10446)
13.	Phytyl tetradecanoate (CID 14486554)
14.	1,5‐diphenyl‐2h‐1,2,4‐triazoline‐3‐thione (CID 2802516)
15.	13‐docosen‐1‐ol, (z)‐ (CID 5354168)
16.	Eicosen‐1‐ol, cis‐9‐ (CID 5364523)
17.	N‐hexadecanoic acid (CID 985)
18.	Phytyl palmitate (CID 6437053)
19.	L‐(+)‐ascorbic acid 2,6‐dihexadecanoate (CID 54722209)
20.	Bis[di(trimethylsiloxy) phenylsiloxy] trimethylsiloxyphenylsiloxane (CID 6422911)
21.	Z,z‐6,27‐hexatriactontadien‐2‐one (CID 5364674)
22.	Hexadecanoic acid, 1‐(hydroxymethyl)‐1,2‐ethanediyl ester (CID 99931)
23.	Cyclotrisiloxane, 2,4,6‐trimethyl‐2,4,6‐triphenyl‐ (CID 18901)

### 3.2. Acute Toxicity studies

In our previous study, the acute toxicity of EaNFL was evaluated at different doses (200, 400, 1000, 2000, 3000, and 4000 mg/kg body weight). No visible signs of toxicity or mortality were observed during the 14‐day observation period. These findings indicate that the LD₅₀ (median lethal dose) of the extract is greater than 4000 mg/kg body weight, suggesting a favorable safety profile [48].

### 3.3. Anxiolytic activity study

#### 3.3.1. EPM

**Figure 1:**
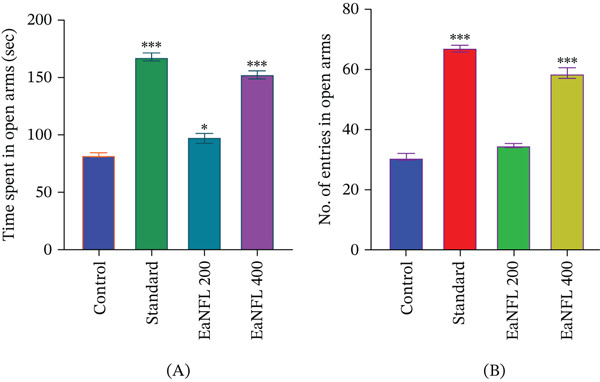
Effects of EaNFL on EPM. (A) Time spent in the open arms and (B) The number of entries in the open arms. The results were indicated as mean ± SEM,  ^∗∗∗^ *p* < 0.001,  ^∗∗^ *p* < 0.01,  ^∗^ *p* < 0.05 were considered statistically significant compared to the control group.

EaNFL at doses of 200 and 400 mg/kg (p.o.) significantly increased both the number of open arm entries and the time spent in the open arms in the EPM test compared with the control group (*p* < 0.05). These observations suggest anxiolytic‐like effects, with the 400‐mg/kg dose producing responses qualitatively similar to diazepam (1 mg/kg, i.p.). Among all treated groups, EaNFL at 400 mg/kg and diazepam showed the most pronounced anxiolytic responses (Figure 1A,B), demonstrating a dose‐dependent effect of EaNFL. However, extract also reduced general locomotor activity, which may have influenced exploration patterns and arm entries. Therefore, there is a possibility that, anxiolytic‐like effects may be partially confounded by altered locomotion. A limitation of the present study is that detailed locomotor parameters, such as total distance traveled, speed, and arm entries, were not recorded, which may limit the ability to fully distinguish anxiety‐ or depression‐related effects from general motor suppression.

#### 3.3.2. HBT

**Figure 2:**
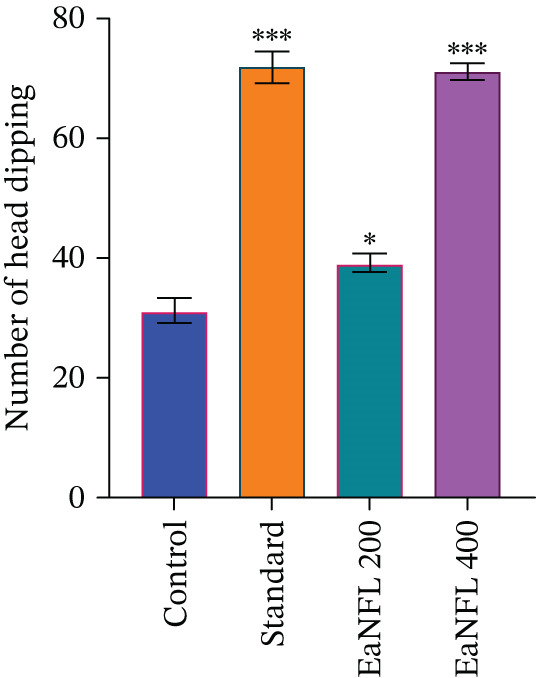
Effects of EaNFL on the HBT in mice. Results are presented as mean ± SEM. Statistical significance relative to the control group was defined as  ^∗∗∗^ *p* < 0.001,  ^∗∗^ *p* < 0.01,  ^∗^ *p* < 0.05.

In the HBT, EaNFL increased head‐dipping behavior in a dose‐dependent manner, with a significant effect observed at 400 mg/kg (*p* < 0.05). This response was comparable with that of diazepam, suggesting notable anxiolytic‐like activity (Figure 2).

### 3.4. Effects of EaNFL on Antidepressant activity test

#### 3.4.1. TST

**Figure 3:**
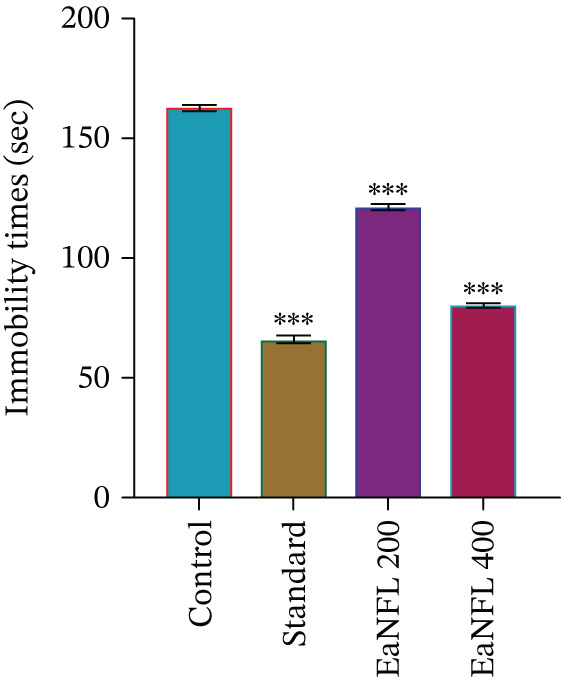
Effect of EaNFL on immobility time in the TST in mice. Values are presented as mean ± SEM. Statistical significance compared to the control group was assessed using  ^∗∗∗^ *p* < 0.001,  ^∗∗^ *p* < 0.01,  ^∗^ *p* < 0.05.

As shown in Figure 3, EaNFL at doses of 200 and 400 mg/kg (p.o.) and imipramine hydrochloride (10 mg/kg, i.p.) significantly reduced the duration of immobility in mice compared with the control group. Moreover, EaNFL exhibited a dose‐dependent effect, with the 400 mg/kg dose producing results quantitatively similar to the reference drug. However, since the extract also reduced general locomotor activity, these observations may be influenced in part by reduced overall activity rather than solely by antidepressant‐like effects.

#### 3.4.2. FST

**Figure 4:**
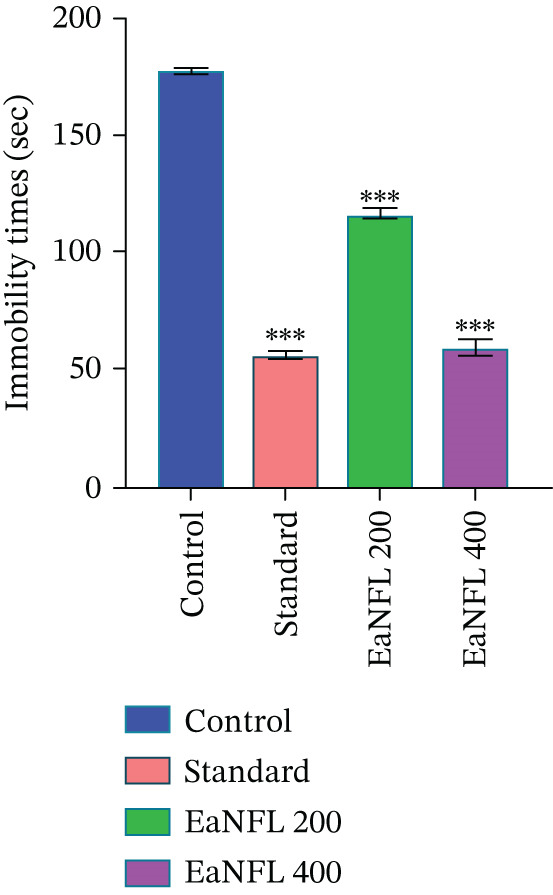
Effects of EaNFL in FST in mice. Results were expressed as mean ± SEM,  ^∗∗∗^ *p* < 0.001,  ^∗∗^ *p* < 0.01,  ^∗^ *p* < 0.05 were considered statistically significant compared with the control group.

In the FST, oral administration of EaNFL at doses of 200 and 400 mg/kg led to a dose‐dependent decrease in immobility duration when compared with the control group, as illustrated in Figure 4 The effect observed with EaNFL at 400 mg/kg was closest to that of imipramine (10 mg/kg), which showed the most pronounced reduction in immobility. As with the TST, reduced locomotor activity may have contributed to these outcomes, and the behavioral effects should therefore be considered indicative of potential antidepressant‐like activity rather than definitive proof of efficacy.

### 3.5. Effects of EaNFL on locomotor activity study (Sedative activity)

#### 3.5.1. OFT

**Figure 5:**
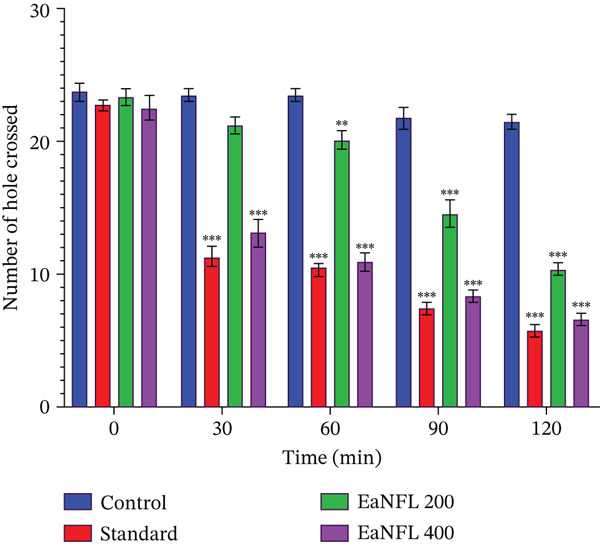
Effect of EaNFL on the OFT. Locomotor activity was assessed by recording the number of squares crossed at various time intervals (0, 30, 60, 90, and 120 min). Data are expressed as mean ± standard error of the mean (SEM). Statistical significance was determined using the following thresholds:  ^∗∗∗^ *p* < 0.001,  ^∗∗^ *p* < 0.01,  ^∗^ *p* < 0.05.

Compared with the control group, mice treated with EaNFL at doses of 200 and 400 mg/kg (p.o.), as well as the reference drug diazepam (1 mg/kg, i.p.), demonstrated a significant reduction in general locomotor activity, as indicated by the number of squares crossed during the final four observation intervals (30, 60, 90, and 120 min), as shown in Figure 5. Notably, the effect observed with the 400‐mg/kg dose of EaNFL was comparable with that of diazepam.

#### 3.5.2. HCT

**Figure 6:**
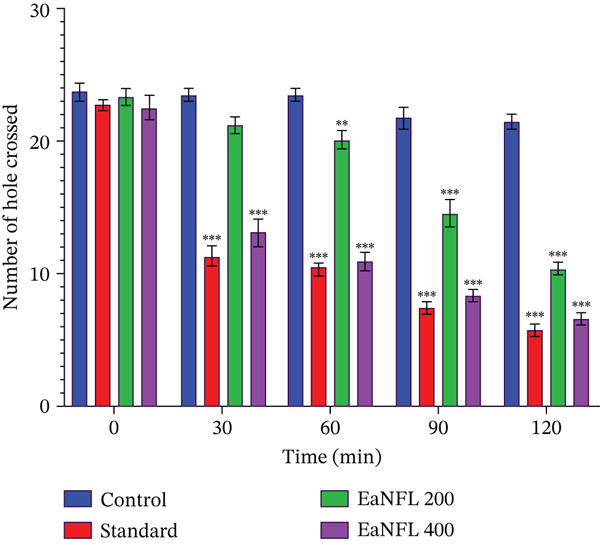
Effect of EaNFL on the HCT. The number of holes crossed was recorded at various time intervals (0, 30, 60, 90, and 120 min). Data are presented as mean ± standard error of the mean (SEM). Statistical significance was considered at  ^∗∗∗^ *p* < 0.001,  ^∗∗^ *p* < 0.01,  ^∗^ *p* < 0.05 compared with the control group.

In the HCT, treatment with EaNFL at both 200 and 400 mg/kg (p.o.) resulted in a significant reduction in the number of holes crossed. This inhibitory effect began at 30 min and persisted throughout the 120‐min observation period compared with the control group. Similarly, the reference drug diazepam (1 mg/kg, i.p.) produced comparable results, as shown in Figure 6. Notably, at the 30‐min interval, the 400‐mg/kg dose of EaNFL and diazepam significantly reduced the number of holes traversed compared with the 200‐mg/kg EaNFL group.

EaNFL at 200 mg/kg produced a mild increase in head‐dip counts, whereas 400 mg/kg significantly increased head dipping compared with the control group (∗*p* < 0.05). The effect at 400 mg/kg was comparable with diazepam, suggesting significant anxiolytic‐like activity.

Because the extract reduced locomotor activity, the possibility that general central nervous system (CNS) suppression contributed to the observed behavioral effects cannot be excluded, and anxiety‐ and depression‐related interpretations should therefore be made cautiously. Future studies incorporating motor control experiments and locomotion‐independent endpoints will be necessary to further clarify the behavioral specificity of these effects.

### 3.6. In Silico study

#### 3.6.1. ADMET analysis

ADMET profiling of identified 23 phytochemicals identified (‐)‐epicatechin, caffeic acid, rosmarinic acid, rutin, kaempferol, quercetin, and myricetin as compounds with favorable pharmacokinetic properties and low toxicity, exhibiting LD50 values between 1.565 and 2.811 and no significant hormonal, acute, or carcinogenic effects. These findings highlight epicatechin, caffeic acid, rosmarinic acid, quercetin, kaempferol, and myricetin as promising candidates for further study. Comprehensive ADME and toxicity data are presented in Tables S1 and S2.

#### 3.6.2. MD analysis

Compounds meeting the predefined selection criteria were subjected to MDS in complex with the target proteins 5I6X, 4UUJ, and 6X3X. Detailed MD results, including binding affinities and key interactions, are presented in Tables S3, S4, and S5 for each respective target. The docking analysis identified several compounds with binding affinities stronger than −7.0 kcal/mol, as summarized in Table 2. For the 5I6X protein, compounds CID 5280343, 5280863, and 5281672 exhibited notable binding affinities of −8.3, −8.2, and −9.7 kcal/mol, respectively, compared with the reference compound imipramine (−6.8 kcal/mol). In the case of 4UUJ, CID 5280343, 5281672, and 5281792 showed binding affinities of −7.7, −8.0, and −7.7 kcal/mol, respectively, outperforming the control diazepam (−5.1 kcal/mol). For 6X3X, strong binding was observed with compounds CID 444539, 5281672, 5280343, and 72276, exhibiting binding affinities of −8.6, −8.8, −8.1, and −8.1 kcal/mol, respectively, whereas diazepam showed a binding affinity of −7.8 kcal/mol. These findings suggest that several screened compounds consistently demonstrated relative binding potential compared with standard drugs across all three targets, underscoring their potential as promising multi‐target therapeutic candidates.

**Table 2:** Binding affinities of selected compounds with 5I6X, 4UUJ, and 6X3X.

SN	PubChem ID	Phytochemical’s name	Docking score (kcal/mol)		
			5I6X	4UUJ	6X3X
1.	**3016 (control)**	Diazepam	**—**	**−5.1**	**−7.8**
2.	**3696 (control)**	Imipramine hydrochloride	**−6.8**	**—**	**—**
3.	5280805	Rutin	−10.2	−9.4	−8.8
**4.**	**5281672**	**Myricetin**	−9.7	−8.0	−8.8
**5.**	**5280343**	**Quercetin**	−8.3	−7.7	−8.1
**6.**	**5280863**	Kaempferol	−8.2	−7.6	−7.6
7.	72276	Epicatechin	−8.0	−7.5	−8.1
8.	5281792	Rosmarinic Acid	−7.8	−7.7	−7.5
9.	530421	Aristolene	−7.6	−6.8	−6.9
10.	2802516	1,5‐Diphenyl‐1H‐1,2,4‐triazole‐3(2H)‐thione	−7.4	−6.0	−7.9
11.	227829	Guaiol	−7.3	−6.6	−7.7
12.	444539	Cinnamic Acid	—	—	−8.6

*Note:* Bold values data present the highest value of the control and highlight the ligands with the highest binding affinity that are considered as lead compounds.

#### 3.6.3. Analysis of Protein–Ligand interaction profiles

The interpretation of protein–ligand interactions focuses on identifying and characterizing the noncovalent contacts such as hydrogen bonds and hydrophobic interactions, which stabilize the ligand within the binding pocket and influence affinity and specificity [49]. Quantitative analyses often involve mapping interaction fingerprints and assessing energetic contributions to pinpoint key residues for ligand optimization [50]. These insights are instrumental in rational drug design, as they help determine which structural modifications may enhance binding strength or selectivity.

**Figure 7:**
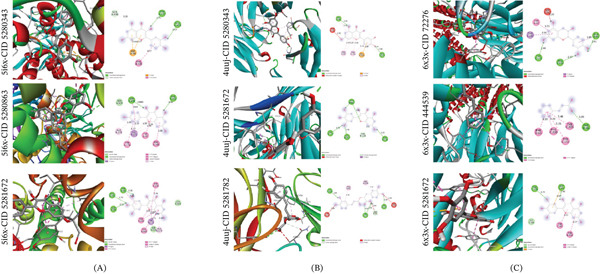
(A–C) (A) 2D and 3D interaction diagrams of the protein–ligand complexes for the 5I6X protein. (B) 2D and 3D interaction diagrams of the protein–ligand complexes for the 4UJJ protein. (C) 2D and 3D interaction diagrams of the protein–ligand complexes for the 6X3X protein.

In the 4UUJ complex, myricetin (CID: 5281672) forms four hydrogen bonds with residues VAL115, LYS207, THR142, and ASN138. Similarly, rosmarinic acid (CID: 5281792) forms three hydrogen bonds with VAL114, TYR95, and VAL155, while quercetin (CID: 5280343) forms two hydrogen bonds with VAL93 and ALA173. The 2D and 3D interaction diagrams for the 4UUJ–ligand complexes are shown in Figure 7A. For the 5I6X complex, quercetin interacts through three hydrogen bonds with TYR175, GLU493, and SER438. Kaempferol (CID: 5280863) forms two hydrogen bonds with SER438 and PHE335, while myricetin establishes three hydrogen bonds with TYR95, PHE335, and ASP98. Corresponding 2D and 3D interaction figures for the 5I6X–ligand complexes are presented in Figure 7B. In the 6X3X complex, epicatechin (CID: 72276) forms three hydrogen bonds with ALA45, ARG114, and TYR172. Cinnamic acid (CID: 444539) forms a single hydrogen bond with PHE46, whereas myricetin forms two hydrogen bonds with THR176 and ASP43. The interaction diagrams for the 6X3X–ligand complexes are provided in Figure 7C.

Multiple well‐oriented hydrogen bonds indicate strong and specific ligand–protein interactions. Ligands forming stable hydrogen bonds with conserved active‐site residues are likely to exhibit higher binding affinity and greater therapeutic potential, as illustrated in Figure 7 and summarized in Table 3.

**Table 3:** Hydrogen bonding and other key interactions in protein–ligand complexes.

Complexes	Hydrogen bond interactions	Other bond interactions
4ujj – CID 5280343	VAL 93, ALA 173	THR 115, PRO 41, PRO 154, GLU 153
4ujj – CID 5281672	VAL 115, LYS 207, THR 142, ASN 138	THR 114
4ujj – CID 5281792	VAL 114, TYR 95, VAL 155	THR 115, PRO 41, PRO 154, ASN 41, THR 156
5i6x – CID 5280343	TYR 175, GLU 493, SER 438	ASP 98, PHE 335
5i6x – CID 5280863	SER 438, PHE 335	ALA 173, TYR 176, ILE 172, TYR 95, PHE 341
5i6x – CID 5281672	TYR 95, PHE 335, ASP 98	TYR 176, SER 438, GLY 442, ILE 172, ALA 173
6x3x – CID 72276	ALA 45, ARG 114, TYR 172	SER 217, TYR 62, THR 215
6x3x – CID 444539	PHE 46	TYR 205, TYR 157, PHE 200, PHE 65
6x3x – CID 5281672	THR 176, ASP 43	TYR 62, TYR 210

#### 3.6.4. MDS

MDS is a useful tool in post‐dock analysis since they allow exploration of time‐dependent stability and atom movement [51]. In MDS, RMSD, RMSF, Rg, SASA, PSA, and H‐bond interactions provide a comprehensive understanding of the behavior of protein–ligand complexes [52]. Compounds CID 5280343, 5281672, and 5281792 along with control drugs were selected to conduct MDS complexed with 5I6X, 4UUJ, and 6X3X protein.

**Figure 8:**
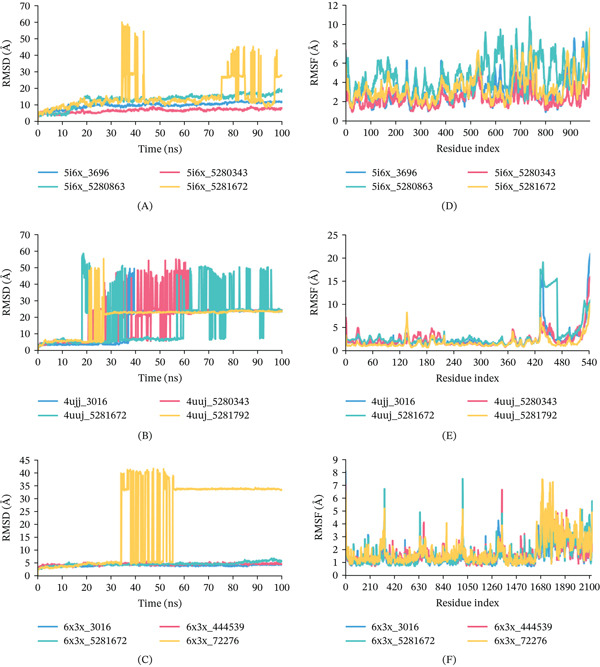
RMSD values calculated from ligand atoms and RMSF values from protein C*α* atoms of the docked protein–ligand complexes during a 100‐ns MDS: (A) Ligand RMSD of 5I6X, (B) Ligand RMSD of 4UUJ, (C) Ligand RMSD of 6X3X, (D) Protein C*α* RMSF of 5I6X, (E) Protein C*α* RMSF of 4UUJ, and (F) Protein C*α* RMSF of 6X3X.

RMSD analysis: As demonstrated in Figure 8, the RMSD of the docked complexes were assessed to evaluate the rigidity and stability of the protein–ligand complexes during the simulation period. For 5I6X, 5I6X + CID 5280343 (Red Color), 5I6X + CID 5280863 (Green Color), 5I6X + CID 5281672 (Yellow Color), and CID 3696 (Control) (Blue Color), the average C*α*‐RMSD values were 6.80, 13.21, 15.40, and 10.01 Å, respectively (Figure 8A). As of 4UUJ, 4UUJ + CID 5280343, 4UJJ + CID 5281672,4UJJ + CID 5281792, and 4UJJ + CID 3016 (Control), the average C*α*‐RMSD values were 20.34, 17.29, 18.89, and 16.80 Å (Figure 8B). Notably, CID 5281672 complex exhibited slight fluctuations during the simulation, and others maintaining an equilibrium state throughout. For 6X3X, 6X3X + CID 444539, 6X3X + CID 5281672, 6X3X + CID 72276, and 6X3X + CID 3016 (Control), the average C*α*‐RMSD values were 4.54, 4.64, 21.09, and 4.05 Å (Figure 8C).

RMSF analysis: RMSF is used to measure how much different parts of a protein fluctuate during interactions with other molecules. For the protein 5I6X (Figure 8D), the average displacements when associated with compounds CID 5280343, 5280863, 5281792, and 3696 (Control) were 2.28, 4.62, 3.36, and 3.14 Å, respectively. The complex with CID 5280863 exhibited greater mobility in certain regions than in others. The average displacements for protein 4UUJ with compounds CID 5280343, 5281672, 5281792, and 3016 (Control) were 2.50, 3.51, 1.82, and 2.59 Å, respectively in Figure 8E. Significantly, the CID 5281672 complex exhibited marginally more mobility between residues 431 and 469, in contrast to the other two complexes. In summary, 4UUJ and its complexes exhibited minimal movement, indicating a high degree of stability. For 6X3X, 6X3X + CID 444539, 6X3X + CID 5281672, 6X3X + CID 72276, and 6X3X + CID 3016 (Control), the average RMSF values were 1.63, 1.56, 1.80, and 1.50 Å (Figure 8F).

**Figure 9:**
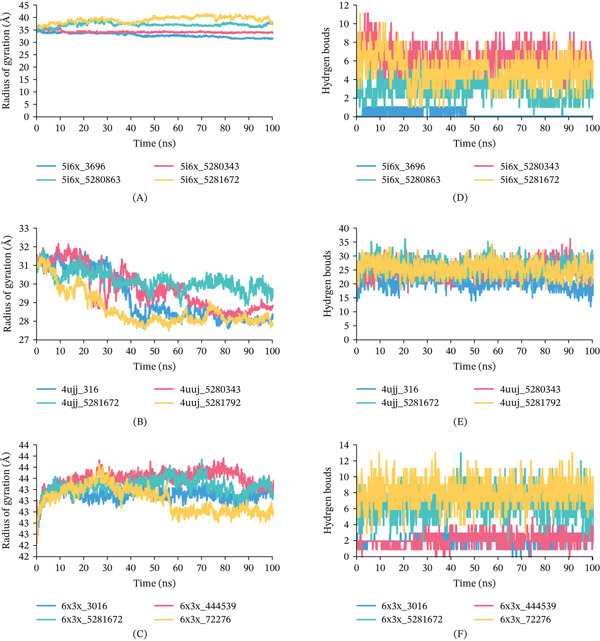
Rg and H‐ bond formation profiles of protein–ligand complexes during a 100‐ns MDS: (A) Rg of 5I6X, (B) Rg of 4UUJ, (C) Rg of 6X3X, (D) hydrogen bonds of 5I6X, (E) hydrogen bonds of 4UUJ, and (F) hydrogen bonds of 6X3X.

Rg analysis: The Rg can be calculated by using the protein’s center of mass, which indicates the degree of structural compactness. The protein will change over time if it is unstable; if it is stable, it will stay constant. The complexes of CID 5280343, 5281672, 5281792, 3696 with 5I6X, 4UUJ, and 6X3X protein, which were notably demonstrated small variations in our analysis, suggesting their stability over time, illustrated in Figures 9A, 9B, and 9C. The Rg values for 5I6X + CID 5280343, 5I6X + CID 5280863, 5I6X + CID 5281672, and 5I6X+ CID 3696 complexes revealed an average of 34.05, 36.84, 38.83, and 32.71 Å correspondingly. As for the 4UUJ + CID 5280343, 4UUJ + CID 5281672, 4UUJ + CID 5281792, and 4UJJ + CID 3016 (Control) complexes, the average Rg values were 29.71, 30.16, 28.67, and 29.30 Å, respectively. As for the 6X3X, 6X3X + CID 444539, 6X3X + CID 5281672, 6X3X + CID 72276, and 6X3X+ CID 3016 (Control), the average Rg values were 43.59, 43.45, 43.22, and 43.32 Å, respectively. Throughout the simulation’s runtime, all protein complexes demonstrated a continuously steady trajectory without notable deviations.

Hydrogen bond interactions: H‐bond analysis of protein–ligand complexes were analyzed via 100 ns simulation time. The hydrogen bonds number for all complexes are represented in Figure 9D, 9E, and 9F. The hydrogen bonds count for 5I6X + CID 5280343, 5I6X + CID 5280863, 5I6X + CID 5281672, and 5I6X+ CID 3696 complexes revealed 11, 6, 11, and 1 correspondingly. As for the 4UUJ + CID 5280343, 4UUJ + CID 5281672, 4UUJ + CID 5281792, and 4UJJ + CID 3016 (Control) complexes, the hydrogen bonds numbers reached 36, 36, 34, and 28, respectively, across the simulation time. As for the 6X3X, 6X3X + CID 444539, 6X3X + CID 5281672, 6X3X + CID 72276, and 6X3X + CID 3016 (Control), the hydrogen bonds contacts reached 5, 13, 13, and 4, respectively.

**Figure 10:**
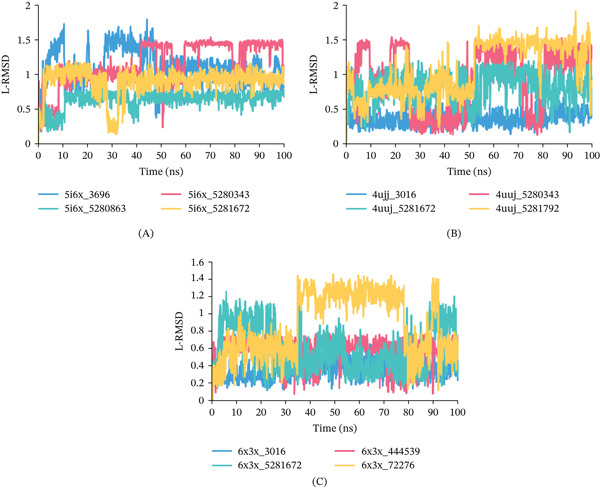
L‐RMSD of the complexes during a 100‐ns MDS: (A) L‐RMSD of 5I6X, (B) L‐RMSD of 4UUJ, (C) L‐RMSD of 6X3X.

Ligand Fit protein RMSD analysis: Figure 10 illustrates the ligand RMSD (L‐RMSD) profiles were analyzed to evaluate the positional stability of the ligands within the binding pockets throughout the 100‐ns simulation. For 5I6X complexes, 5I6X + CID 5280343 (Red), 5I6X + CID 5280863 (Green), 5I6X + CID 5281672 (Yellow), and the control CID 3696 (Blue) exhibited L‐RMSD values generally within 0.2–1.6 Å (Figure 10A). Among them, CID 5280863 maintained comparatively lower fluctuations (0.5–0.8 Å), whereas CID 5280343 showed transient increases near 1.4–1.5 Å during the mid‐simulation phase. For 4UUJ complexes, 4UUJ + CID 5280343, 4UUJ + CID 5281672, 4UUJ + CID 5281792, and the control CID 3016 demonstrated L‐RMSD variations within 0.2–1.7 Å (Figure 10B). The control ligand CID 3016 remained relatively stable (0.3–0.5 Å), while CID 5281792 showed comparatively higher fluctuations approaching 1.5–1.7 Å toward the later simulation stages. For 6X3X complexes, 6X3X + CID 444539, 6X3X + CID 5281672, 6X3X + CID 72276, and the control CID 3016 displayed L‐RMSD values ranging approximately from 0.2 to 1.4 Å (Figure 10C), with CID 72276 exhibiting the highest deviations during the mid‐trajectory period.

**Figure 11:**
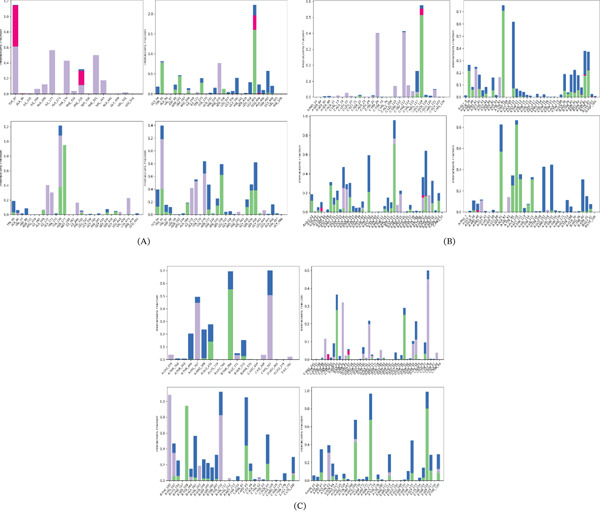
PL‐Contacts of the complexes during a 100‐ns MDS: (A) PL‐Contacts of all compounds with 5I6X, (B) PL‐Contacts of all compounds 4UUJ, (C) PL‐Contacts of all compounds 6X3X.

P‐L contact: Protein–ligand interaction profiles were analyzed to identify key residues contributing to ligand binding stability during the 100‐ns simulation (Figure 11). In the 5I6X complexes (Figure 11A), the control ligand CID 3696 did not form persistent hydrogen bonds throughout the simulation. In contrast, CID 5280343 formed multiple hydrogen bond interactions with TYR95, ASP98, ASN101, ARG104, TYR175, SER336, GLU493, THR497, and GLY498. CID 5280863 showed hydrogen bonding with TYR95, ALA169, TYR176, ASN177, PHE335, SER438, and GLY442, while CID 5281672 interacted with GLY94, TYR95, ALA169, ASN177, PHE335, SER336, GLY435, SER439, and GLY442, indicating stable contacts within the active site. For 4UUJ complexes (Figure 11B), the control ligand CID 3016 interacted with SER22, ARG117, and GLU118. CID 5280343 formed hydrogen bonds with GLN39, ARG40, GLY42, SER91, TYR95, ALA110, THR40, ASN41, SER43, and ASN85, among others. CID 5281672 interacted with GLU62, GLN65, ALA130, PRO131, GLY132, ALA134, GLN136, MET140, THR142, SER116, and ASN210, whereas CID 5281792 showed interactions with GLN39, SER91, TYR95, ALA110, GLY111, VAL114, THR115, and GLY42. In the 6X3X complexes (Figure 11C), the control ligand CID 3016 interacted with ARG269, GLU270, THR268, and SER272. CID 444539 formed hydrogen bonds with ASP44, ARG67, THR197, HIS218, and GLU27. CID 5281672 interacted with HIS102, LYS156, SER159, ALA161, ASP43, ILE44, GLN64, and LYS180, while CID 72276 showed interactions with ASP43, GLN64, ARG169, ASN173, ARG114, TYR172, THR215, THR216, SER217, and TYR220, indicating strong and persistent contacts within the binding pocket.

**Figure 12:**
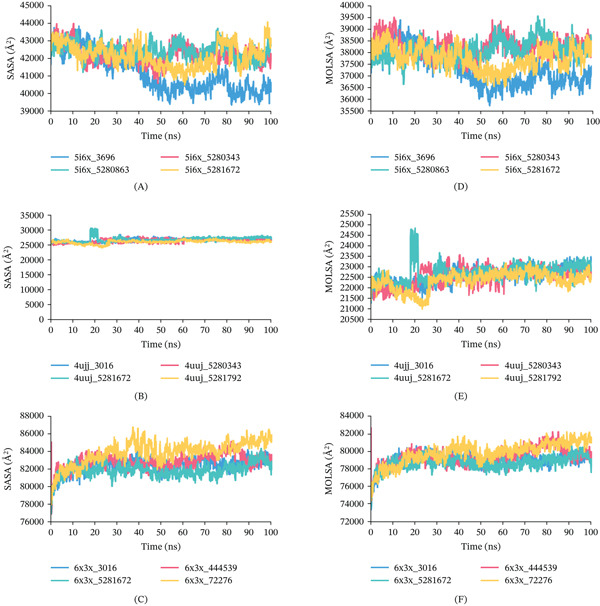
Graphical representation of SASA and MolSA of the protein–ligand complexes over a 100‐ns MDS: (A) SASA for 5I6X, (B) SASA for 4UUJ, (C) SASA for 6X3X, (D) MolSA for 5I6X, (E) MolSA for 4UUJ, and (F) MolSA for 6X3X.

SASA analysis: The SASA of all the complexes were undertaken to examine whether there had been any alterations to the protein surface or volume. The total SASA that reflects the area of the bimolecular surface that solvent molecules can reach. Protein volumes are correlated with a lower SASA profile and a greater SASA profile, respectively. For 5I6X, 5I6X + CID 5280343, 5I6X + CID 5280863, and 5I6X + CID 5281672 complex displayed almost constant SASA trajectory defined by modest changes during the 100‐ns simulation time, presented in Figures 12A, 12B, and 12C. In the case of the 4UUJ protein, CID 5281672 displayed a little fluctuation rather than CID 5280343, and 5281792 shows consistent SASA. In case of 6X3X, all complexes show greater SASA values that demonstrate a longer protein volume.

MolSA analysis: The MolSA represents the total surface area of a molecule calculated using a defined probe radius, encompassing both polar and non‐polar regions. For the 5I6X protein and its complexes with CID 5280343, 5280863, and 5281672, the MolSA values remained relatively stable, displaying only minor fluctuations over the 100‐ns simulation period, as illustrated in Figures 12D, 12E, and, 12F. Similarly, the 4UUJ complexes comprising CID 5280343, 5281672, 5281792, and the control compound CID 3016 exhibited a consistent MolSA trajectory, with CID 5281672 showing a slight deviation. In the case of 6X3X and its associated complexes with CID 444539, 5281672, 72276, and the control CID 3016, the MolSA values remained nearly constant throughout the simulation, suggesting stable molecular surface conformation across all complexes.

**Figure 13:**
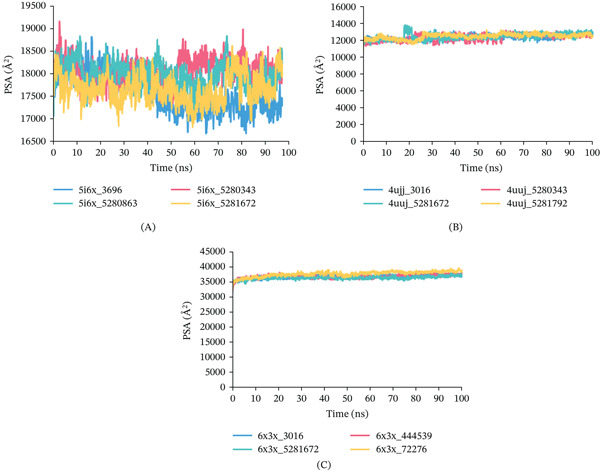
Graphical representation of PSA fluctuations of the protein–ligand complexes over a 100‐ns MDS: (A) PSA for 5I6X, (B) PSA for 4UUJ, (C) PSA for 6X3X.

PSA analysis: The PSA analysis provides critical insights into the dynamic behavior of protein surfaces during MDS. PSA refers to the portion of the SASA contributed exclusively by polar atoms, specifically oxygen and nitrogen, within a molecule. For 5I6X, 5I6X + CID 5280343, 5I6X + CID 5280863, and 5I6X + CID 5281672 complex displayed minor fluctuation during the 100‐ns simulation time, presented in Figures 13A, 13B, and, 13C. As for the 4UUJ + CID 5280343, 4UUJ + CID 5281672, 4UUJ + CID 5281792, and 4UJJ + CID 3016 (Control) complexes, PSA remains almost flat except CID 5281672 during the simulation time. As for the 6X3X, 6X3X + CID 444539, 6X3X + CID 5281672, 6X3X + CID 72276, and 6X3X + CID 3016 (Control), all complex shows constant PSA trajectory.

## 4. Discussion

Since ancient times, people have utilized plants for therapeutic purposes, and many current drugs are either inspired by or derived from the chemical substances found in plants. The ability of plants to produce a large variety of physiologically active chemicals that can be utilized to treat a wide range of disorders accounts for their significance in pharmacology [53]. Natural products obtained from medicinal plants provide a vast reservoir of bioactive chemicals with varied pharmacological properties, such as anti‐inflammatory, antioxidant, anxiolytic, antidepressant, and neuroprotective effects [54]. The therapeutic potential of phytochemicals lies in their structural diversity and capacity to interact with multiple molecular targets, making them particularly valuable in addressing complex neuropsychiatric conditions such as anxiety and depression. These conditions frequently include multiple dysregulated neurotransmitter systems, including GABAergic, serotonergic, and dopaminergic pathways, making single‐target synthetic drugs sometimes ineffective or may have more side effects [55]. On the other hand, plant extracts may provide a balanced regulation of these pathways with improved safety profiles due to their natural origin and reduced toxicity [56].

In recent years, research has been focused on identifying novel phytochemicals with CNS action. Flavonoids, alkaloids, terpenes, and phenolic acids are among the most extensively researched phytoconstituents due to their anxiolytic, antidepressant, and sedative characteristics [57]. In this regard, the study of *N. fruticans*, a mangrove palm with antioxidant and anti‐inflammatory properties, provides a chance to look into its CNS‐modulating effects. *NF* is known for its high concentration of polyphenols, flavonoids, vitamin E, protocatechuic acid, chlorogenic acid, kaempferol, and calcium, and it is gaining popularity for its numerous health advantages. The phytochemical analysis identified many elements, including as alkaloids, carbohydrates, tannins, glycosides, saponins, flavonoids, phenolic compounds, triterpenes, and gum, with antioxidant and biological characteristics [48]. The phytochemical composition of plant extracts may clarify their medicinal and physiological functions [58]. Numerous well‐known medicinal herbs have antidepressant and/or anxiety‐relieving qualities. Although *NF* has traditionally been employed in the treatment of various ailments, its pharmacological impact on the CNS remains unexplored. This study aims to assess the neuropharmacological properties of EaNFL on the CNS by employing computational approaches to identify the most promising bioactive candidates, specifically targeting the gamma‐aminobutyric acid type A receptor (GABA_A_; 6X3X), a potassium channel blocker (4UUJ), and the SERT (5I6X).

Preliminary phytochemical analysis of EaNFL revealed a diverse range of bioactive compounds. HPLC analysis identified high levels of (−)‐epicatechin, along with other phenolics such as trans‐cinnamic acid, rutin hydrate, quercetin, caffeic acid, kaempferol, myricetin, and rosmarinic acid—all known for their potent antioxidant and anti‐inflammatory properties. Additionally, GC‐MS analysis detected 15 more phytochemicals with recognized therapeutic potential, underscoring the extract’s pharmacological significance [31].

The current experimental design suggests the presence of anxiolytic, antidepressant‐like, and sedative effects in EaNFL. The EPM and HBT are well‐known models for determining anxiolytic behavior in rodents. In the EPM, anxiolytic action is indicated by a considerable increase in both times spent and number of entries into open arms [59]. The observed suppression of locomotor activity and sedative effects of EaNFL revealed by HCT and OFT, suggest a possible CNS‐depressant action. While benzodiazepines traditionally function via GABA_A_ receptor‐mediated inhibition, the anxiolytic‐like effects of EaNFL observed here suggest a promising parallel that may involve similar GABAergic modulation. These findings provide a compelling basis for future pharmacological and biochemical studies to further characterize this specific mechanism. Activation of GABA_A_ receptors are known to reduce neuronal excitability, thereby contributing to its calming effects [60, 61].

Our experimental findings demonstrate that EaNFL produced dose‐dependent anxiolytic and sedative effects, with the 400 mg/kg dose exhibiting efficacy that aligns with diazepam. As illustrated in Figures 1 and 2, enhanced behavioral responses and a significant increase in head‐dip frequency in the HBT suggest its anxiolytic activity. These effects may be attributed to the presence of flavonoids and other polyphenolic compounds in EaNFL, which are known to interact with the benzodiazepine‐binding site of GABA_A_ receptors, potential possibility of mimicking the mechanism of action of standard anxiolytics like diazepam [62].

The FST and TST are well‐established behavioral models for evaluating antidepressant activity in mice, with reduced immobility time reflecting an antidepressant‐like effect. This behavioral outcome is associated with enhanced serotonergic, noradrenergic, and dopaminergic neurotransmission. Conventional antidepressants, such as tricyclics, SSRIs, and MAOIs, achieve their effects by inhibiting the reuptake of serotonin and NE and modulating 5‐HT and adrenergic receptors. In this study, the reference drug imipramine acted by increasing synaptic levels of both neurotransmitters. Additionally, dopamine signaling particularly via D_1_ and D_2_ receptors has been implicated in mediating antidepressant responses, as supported by preclinical evidence [61, 63]. According to Scapagnini et al. [64], antioxidants are known to modulate SERT activity, potentially eliciting antidepressant‐like effects. In this study, EaNFL treatment at 200 and 400 mg/kg significantly reduced immobility time in both the FST and TST (Figures 4 and 5), with the higher dose showing effects comparable with diazepam. These behavioral observations suggest antidepressant potential, likely mediated by enhanced serotonergic transmission through SERT inhibition a recognized mechanism of action for many standard antidepressants [65]. But further biochemical and molecular studies are required to definitively characterize the underlying pathway.

The OFT and HCT were used to evaluate the locomotor activity and sedative potential of EaNFL. In the OFT (Figure 5), the number of squares crossed by mice decreased in a dose‐dependent manner, with a marked reduction at 400 mg/kg, indicating diminished spontaneous motor activity and potential sedative effects. Similarly, in the HCT (Figure 6), mice treated with 200 and 400 mg/kg of EaNFL showed a significant decrease in hole crossings compared with the control group, with the higher dose producing effects comparable with the standard sedative, diazepam. These findings suggest that EaNFL exhibits CNS depressant properties, possibly through enhancement of GABAergic transmission and suppression of excitatory neuronal activity [66]. Additionally, modulation of potassium ion channels, which regulate neuronal excitability [67], may contribute to the observed reduction in locomotor activity. While the exact mechanisms remain to be fully elucidated, the polyphenols and flavonoids identified in EaNFL suggest a possible basis for the observed sedative and anxiolytic‐like activities.

Although EaNFL reduced locomotor activity in the OFT and HCT, the significant increase in open‐arm exploration in the EPM, enhanced head‐dipping behavior in the HBT, and decreased immobility in the TST and FST indicate that its anxiolytic and antidepressant‐like effects are not solely due to sedation but likely involve genuine neurobehavioral modulation. The constituents of EaNFL may exert antidepressant, anxiolytic, and locomotor‐suppressing effects through modulation of key neurotransmitter systems. Notably, polyphenols and alkaloids identified in the extract have been shown in previous studies to inhibit serotonin and NE transporters and exhibit affinity for GABA_A_ receptors, mechanisms that are closely associated with the regulation of mood, anxiety, and motor activity [61].

The results of this study suggest that several phytochemicals within EaNFL may possess anxiolytic and antidepressant properties, as indicated by both behavioral assays and predictive in silico modeling. The behavioral assays demonstrate the neuropharmacological activity of EaNFL, whereas the computational analyses provide a predictive framework to explore possible molecular interactions between the identified phytochemicals and CNS‐related targets.

ADMET evaluations indicated that quercetin, myricetin, and rosmarinic acid possess favorable pharmacokinetic profiles, including efficient intestinal absorption and low predicted toxicity, consistent with previous reports supporting their safety [68]. To identify drug candidates from multifarious sources, computer‐aided drug design (CADD) has become a buzzword across the world [69]. MD is a crucial technique in CADD, enabling virtual screening of compound libraries to identify protein–ligand complexes with the best binding affinity at least energy [70].

MD results demonstrated that CID 5280343, 5281672, and 5281792 consistently exhibited stronger binding affinities than the standard drugs diazepam and imipramine across all three target proteins 5I6X, 4UUJ, and 6X3X. Notably, myricetin showed a binding affinity of −9.7 kcal/mol with 5I6X, outperforming imipramine (−7.5 kcal/mol), indicating stronger interactions within the benzodiazepine‐like binding pocket. These results are consistent with previous studies reporting the high affinity of flavonoids for GABAergic and monoaminergic receptors, underscoring their potential for multi‐target neuromodulatory activity [71]. The detailed interaction maps revealed that key hydrogen bonds and hydrophobic contacts stabilize these ligands in their respective binding clefts. Notably, rosmarinic acid’s network of hydrogen bonds within 4UUJ’s active site suggests it could modulate both serotonergic and cholinergic pathways, supporting its dual anxiolytic‐antidepressant profile [72].

MDS have become an essential tool for CADD, which analyzes the movements of atoms [73]. The C*α*‐RMSD explains the overall stability of the proteins and its complexes in a biological system where higher values indicate more significant deviations from the starting structure [74]. The findings highlighted the stability and minimal fluctuations within the protein–ligand complexes. Additionally, RMSF analysis offered important information regarding the flexibility and dynamic motions of the complexes, where higher RMSF values correspond to increased residue flexibility, and lower values indicate greater structural stability [75].

For the 5I6X protein (Figure 8A), CID 5280343 exhibited the lowest RMSD value (6.80 Å), indicating greater conformational stability, followed by the control compound CID 3696 (10.01 Å). In the case of 4UUJ (Figure 8B), all ligand–protein complexes showed slightly higher RMSD values compared with the control. Among them, CID 5280343 (20.34 Å) and 5281672 (17.29 Å) demonstrated performance similar to the control CID 3016 (16.80 Å). For 6X3X (Figure 8C), CID 3016 (4.05 Å), 444539 (4.54 Å), and 5281672 (4.64 Å) maintained stable conformations throughout the simulation, whereas CID 72276 exhibited significant fluctuations (21.09 Å), indicating poor binding stability.

RMSF analysis further revealed residue‐level flexibility across the complexes. For 5I6X (Figure 8D), CID 5280343 showed the lowest average fluctuation (2.28 Å), indicating strong and stable binding. In contrast, CID 5280863 (4.62 Å) and 5281792 (3.36 Å) exhibited higher fluctuations than the control CID 3696 (3.14 Å), suggesting increased localized flexibility. In the 4UUJ complex (Figure 8E), CID 5281792 demonstrated the least fluctuation (1.82 Å), while CID 5281672 showed slightly higher flexibility (3.51 Å), particularly in the residue range 431–469, compared with the control CID 3016 (2.59 Å). For the 6X3X system (Figure 8F), all complexes displayed low RMSF values, with CID 5281672 (1.56 Å) showing comparable stability to the control CID 3016 (1.50 Å), whereas CID 72276 (1.80 Å) exhibited slightly greater flexibility.

Rg analysis reflects the compactness and structural stability of protein–ligand complexes. For the 5I6X protein (Figure 9A), CID 5280343 (34.05 Å) and the control, 3696 (32.71 Å) exhibited relatively compact and stable conformations. In contrast, CID 5280863 (36.84 Å) and 5281672 (38.83 Å) showed higher Rg values, suggesting fewer compact structures. In the case of 4UUJ (Figure 9B), all complexes displayed stable profiles, with CID 5281792 exhibiting the greatest compactness (28.67 Å), followed by CID 3016 (29.30 Å), 5280343 (29.71 Å), and 5281672 (30.16 Å), indicating only minor variations in structural compactness. For 6X3X (Figure 9C), the Rg values remained nearly constant across all complexes CID 444539 (43.59 Å), 5281672 (43.45 Å), 72276 (43.22 Å), and control CID 3016 (43.32 Å) demonstrating strong conformational stability throughout the simulation.

H‐bond analysis over a 100‐ns simulation further revealed the strength of protein–ligand interactions. For 5I6X (Figure 9D), CID 5280343 and 5281672 each formed 11 hydrogen bonds, indicating robust binding interactions, whereas CID 5280863 and 3696 (control) formed only 6 and 1, respectively. In the 4UUJ complex (Figure 9E), all ligands formed a high number of hydrogen bonds, with CID 5280343 and 5281672 forming 36 bonds each, CID 5281792 forming 34, and the control CID 3016 forming 28, suggesting stable binding. For 6X3X (Figure 9F), CID 5281672 and 72276 formed 13 H‐bonds each, while CID 444539 and the control CID 3016 formed 5 and 4, respectively. These results indicate that the tested compounds exhibited stronger and more consistent hydrogen bonding interactions compared to the control across all three protein targets.

Surface area analyses including SASA, MolSA, and PSA demonstrated stable conformational behavior across all protein–ligand complexes. For the 5I6X complex, ligands CID 5280343, 5280863, and 5281672 exhibited minimal fluctuations in SASA, MolSA, and PSA values, indicating consistent surface exposure and conformational stability. In the case of the 4UUJ complex, all ligands, CID 5280343, 5281672, 5281792, and the control CID 3016 maintained stable MolSA and PSA profiles, with only a slight deviation observed for CID 5281672. Similarly, for the 6X3X complex, ligands CID 444539, 5281672, 72276, and 3016 (control) demonstrated steady SASA, MolSA, and PSA trajectories, reflecting stable molecular surface and polar exposure throughout the simulation. These results are illustrated in Figures 12A, 12B, and, 12C for SASA, Figures 12D, 12E, and 12F for MolSA, and Figures 13A, 13B, and, 13C for PSA, respectively.

Overall, MDS revealed that myricetin (CID: 5281672), quercetin (CID: 5280343), and (−)‐epicatechin (CID: 72276) exhibited greater conformational flexibility than the control compounds across all target proteins. These results support the structural integrity and stability of the myricetin and quercetin protein complexes under simulated physiological conditions, underscoring their potential in vivo efficacy [51]. The biological activity of EaNFL is likely the result of the combined effects of multiple phytochemicals present in the extract, including both highly active minor constituents and moderately active but more abundant compounds. Given the demonstrated anxiolytic, antidepressant, and locomotor‐modulating activities, the findings of this study may facilitate the development of novel therapeutics for psychiatric disorders.

However, further biochemical, pharmacological, and molecular investigations are warranted to elucidate the underlying mechanisms, including whether the observed effects are mediated by a single bioactive compound or through the synergistic interaction of multiple plant‐derived constituents. Despite these promising findings, several limitations should be acknowledged. The neuropharmacological effects were evaluated exclusively in animal models, limiting their direct translational applicability to humans. Moreover, the study was conducted using a crude ethyl acetate extract of *N. fruticans* leaves, and the specific active constituents were not isolated or experimentally validated. Therefore, future research involving compound isolation, detailed mechanistic studies, and well‐designed clinical trials is required to confirm the therapeutic potential and safety of this extract.

## 5. Conclusion

The findings of the present study suggest that the ethyl acetate extract of *N. fruticans* leaves (EaNFL) possesses notable potential for the treatment of anxiety and depression, supported by compelling neuropharmacological evidence. These effects are primarily attributed to its antioxidant‐rich phytoconstituents, particularly phenols and flavonoids. The results align with the plant’s traditional use in folk medicine, underscoring its ethnopharmacological relevance. Behavioral assays revealed dose‐dependent antidepressant, anxiolytic, and sedative effects, while computational analyses indicated strong binding affinities of several bioactive compounds to key neuroreceptors. Notably, myricetin (CID: 5281672) and quercetin (CID: 5280343) emerged as promising lead compounds due to their multi‐target binding capacities and favorable pharmacokinetic and safety profiles. The integration of in vivo and in silico findings suggest that EaNFL may exert its effects through modulation of serotonergic and GABAergic pathways, supporting its potential as a safer, plant‐based neurotherapeutic agent. Future studies will include locomotion‐independent endpoints to better clarify antidepressant potential. In summary, while the current findings provide provisional evidence of the neuropharmacological potential of EaNFL, these results are preclinical and exploratory. The observed safety profile is limited to acute toxicity test; therefore, chronic toxicity studies and pharmacokinetic study are essential to establish long‐term tolerability. These data serve as a hypothesis‐generating foundation for future research, as further clinical research is required to determine the extract’s therapeutic efficacy and safety margins in humans.

**Nomenclature**
CNS: central nervous system
EaNFL: ethyl acetate extract of *Nypa fruticans* leaves
EPM: elevated plus maze
FST: forced swimming test
GABA: gamma‐aminobutyric acid
GC‐MS: gas chromatography–mass spectrophotometry
HBT: hole board test
HCT: hole cross test
HPLC: high‐performance liquid chromatography
IP: intraperitoneal
MAOIs: monoamine oxidase inhibitors
MD: molecular docking
MDS: molecular dynamics simulation
MolSA: molecular surface area
NE: noradrenaline
NO: nitric oxide
OFT: open field test
PDB: protein data bank
PSA: polar surface area
Rg: radius of gyration
RMSD: root mean square deviation
RMSF: root mean square fluctuation
SASA: solvent accessible surface area
SEM: standard error of the mean
SERT: human serotonin transporter
SID: simulated interaction diagram
SSRIs: selective serotonin reuptake inhibitors
TCA: trichloroacetic acid
TST: tail suspension test

## Author Contributions

**Farhana Islam:** writing – original draft, review and editing, conceptualization, project administration, methodology, visualization, validation, software, resources, investigation, formal analysis, data curation, supervision; **Sabbir Ahmed:** writing – original draft, visualization, software, methodology, validation; **Jannatul Ferdous:** writing – review and editing, software, data curation, formal analysis; **Mostafa Kamal:** formal analysis, software, data curation; **Asma Aktar:** data curation, validation; **Fariya Islam Rodru:** writing – review and editing, formal analysis; **Md. Nurul Islam:** writing – review and editing; **Al Riyad Hasan:** Writing ‐ review and editing, investigation.

## Funding

No funding was received for this manuscript.

## Disclosure

The final manuscript has been read and approved by all authors.

## Conflicts of Interest

The authors declare no conflicts of interest.

## Supporting information


**Supporting Information** Additional supporting information can be found online in the Supporting Information section. Table S1The pharmacokinetic and drug‐likeness profiles of the identified phytochemicals from EaNFL. Key parameters such as molecular weight (MW), hydrogen bond acceptors (NHA), hydrogen bond donors (NHD), lipophilicity (LogP), number of rotatable bonds (NRB), intestinal absorption (IA), total clearance (TC), number of Lipinski’s rule violations (NLV), and drug‐likeness (DL) were evaluated. Most compounds demonstrated favorable pharmacokinetic characteristics with minimal Lipinski’s violations, indicating good oral bioavailability potential. Table S2: The toxicity predictions using Protox 3.0. Parameters including blood–brain barrier (BBB) permeability, oral acute toxicity (LD50), hepatotoxicity (HT), AMES toxicity (AT), maximum tolerated dose (MTD), cytotoxicity (CT), and toxicity class were assessed. Most compounds were predicted to be non‐hepatotoxic and non‐carcinogenic, with relatively safe toxicity classes (mainly class 4–6). Table S3: The molecular docking results of EaNFL‐derived phytochemicals against the target protein (PDB ID: 5I6X), expressed as binding affinity (kcal/mol). Rutin showed the highest binding affinity (−10.2 kcal/mol), followed by myricetin (−9.7 kcal/mol) and quercetin (−8.3 kcal/mol). Other compounds, including kaempferol and epicatechin, also exhibited strong interactions, while the reference drug imipramine showed comparatively lower affinity (−7.5 kcal/mol). Table S4: The docking interactions of EaNFL‐derived phytochemicals with the target protein (PDB ID: 4UJJ). Rutin showed the highest binding affinity (−9.4 kcal/mol), followed by myricetin (−8.0 kcal/mol) and quercetin (−7.7 kcal/mol). Other compounds, including rosmarinic acid and kaempferol, also demonstrated notable interactions, while long‐chain fatty compounds showed weaker binding. Overall, flavonoids exhibited consistent and strong affinity toward the protein’s active site. Table S5: The binding affinities of selected phytochemicals against the target protein (PDB ID: 6X3X). Rutin and myricetin showed the highest affinity (−8.8 kcal/mol), followed by trans‐cinnamic acid (−8.6 kcal/mol). Epicatechin and quercetin also exhibited strong interactions, while the reference drug diazepam showed comparatively lower affinity (−7.8 kcal/mol).

## Data Availability

The data that support the findings of this study are available from the corresponding author upon reasonable request.
