# Supplementary material for: Ameliorative Effects of Nypa fruticans Leaf Extract on Anxiety and Depression: Evidence From In Vivo and In Silico Studies
Source: Biomed Res Int. 2026 Apr 29;2026:1243521. doi: 10.1155/bmri/1243521 (PMC13128989; doi:10.1155/bmri/1243521)
Supplement: Supplementary file 1 — Supporting Information Additional supporting information can be found online in the Supporting Information section. Table S1The pharmacokinetic and drug‐likeness profiles of the identified phytochemicals from EaNFL. Key parameters such as molecular weight (MW), hydrogen bond acceptors (NHA), hydrogen bond donors (NHD), lipophilicity (LogP), number of rotatable bonds (NRB), intestinal absorption (IA), total clearance (TC), number of Lipinski’s rule violations (NLV), and drug‐likeness (DL) were evaluated. Most compounds demonstrated favorable pharmacokinetic characteristics with minimal Lipinski’s violations, indicating good oral bioavailability potential. Table S2: The toxicity predictions using Protox 3.0. Parameters including blood–brain barrier (BBB) permeability, oral acute toxicity (LD50), hepatotoxicity (HT), AMES toxicity (AT), maximum tolerated dose (MTD), cytotoxicity (CT), and toxicity class were assessed. Most compounds were predicted to be non‐hepatotoxic and non‐carcinogenic, with relatively safe toxicity classes (mainly class 4–6). Table S3: The molecular docking results of EaNFL‐derived phytochemicals against the target protein (PDB ID: 5I6X), expressed as binding affinity (kcal/mol). Rutin showed the highest binding affinity (−10.2 kcal/mol), followed by myricetin (−9.7 kcal/mol) and quercetin (−8.3 kcal/mol). Other compounds, including kaempferol and epicatechin, also exhibited strong interactions, while the reference drug imipramine showed comparatively lower affinity (−7.5 kcal/mol). Table S4: The docking interactions of EaNFL‐derived phytochemicals with the target protein (PDB ID: 4UJJ). Rutin showed the highest binding affinity (−9.4 kcal/mol), followed by myricetin (−8.0 kcal/mol) and quercetin (−7.7 kcal/mol). Other compounds, including rosmarinic acid and kaempferol, also demonstrated notable interactions, while long‐chain fatty compounds showed weaker binding. Overall, flavonoids exhibited consistent and strong affinity toward the protein’s a [file BMRI-2026-1243521-s001.docx]

**Supplementary description**

**Table S_1_:** The pharmacokinetic and pharmacophore analysis of phytochemicals from ethyl acetate extract of *Nypa fruticans* leaves (EaNFL)

| **Ligand name** | **MW** | **NHA** | **NHD** | **Log P** | **NRB** | **IA** | **TC** | **NLV** | **DL** |
| --- | --- | --- | --- | --- | --- | --- | --- | --- | --- |
| (-) Epicatechin (CID 72276) | 290.27 | 6 | 5 | 0.85 | 1 | 68.83 | 0.183 | 0 | Yes |
| Caffeic acid (CID 689043) | 180.16 | 3 | 3 | 0.93 | 2 | 69.40 | 0.508 | 0 | Yes |
| Rutin hydrate (CID 5280805) | 628.54 | 16 | 10 | -1.52 | 6 | 25.74 | -0.081 | 3 | No |
| Rosmarinic acid (CID 5281792) | 360.32 | 7 | 5 | 1.52 | 6 | 32.52 | 0.25 | 0 | Yes |
| Myricetin (CID [5281672](https://pubchem.ncbi.nlm.nih.gov/compound/5281672)) | 318.24 | 8 | 6 | 0.79 | 1 | 65.93 | 0.422 | 1 | Yes |
| Kaempferol (CID [5280863](https://pubchem.ncbi.nlm.nih.gov/compound/5280863)) | 286.24 | 6 | 4 | 1.58 | 1 | 74.29 | 0.477 | 0 | Yes |
| Quercetin (CID 5280343) | 302.24 | 7 | 5 | 1.23 | 1 | 77.20 | 0.407 | 0 | Yes |
| trans-Cinnamic acid (CID 444539) | 148.16 | 1 | 1 | 1.79 | 2 | 94.02 | 0.801 | 0 | Yes |
| Guaiol (CID [227829](https://pubchem.ncbi.nlm.nih.gov/compound/227829)) | 222.37 | 1 | 1 | 3.46 | 1 | 93.99 | 1.077 | 0 | Yes |
| (-)-Aristolene (CID [530421](https://pubchem.ncbi.nlm.nih.gov/compound/530421)) | 204.36 | 0 | 0 | 4.37 | 0 | 97.80 | 0.917 | 1 | Yes |
| 2,3-dehydro-4-oxo-.beta.-ionone (CID 5363867) | 204.27 | 2 | 0 | 2.39 | 2 | 97.41 | 0.245 | 0 | Yes |
| Neophytadiene (CID [10446](https://pubchem.ncbi.nlm.nih.gov/compound/10446)) | 278.52 | 0 | 0 | 7.07 | 13 | 92.85 | 1.764 | 1 | Yes |
| Phytyl tetradecanoate (CID [14486554](https://pubchem.ncbi.nlm.nih.gov/compound/14486554)) | 506.9 | 2 | 0 | 10.97 | 26 | 89.26 | 1.776 | 2 | No |
| 1,5-diphenyl-2h-1,2,4-triazoline-3-thione (CID [2802516](https://pubchem.ncbi.nlm.nih.gov/compound/2802516)) | 253.33 | 3 | 1 | 3.18 | 2 | 92.61 | 0.206 | 0 | Yes |
| 13-docosen-1-ol, (z)- (CID [5354168](https://pubchem.ncbi.nlm.nih.gov/compound/5354168)) | 324.59 | 1 | 1 | 7.29 | 19 | 88.25 | 2.12 | 1 | Yes |
| Eicosen-1-ol, cis-9- (CID [5364523](https://pubchem.ncbi.nlm.nih.gov/compound/5364523)) | 296.54 | 1 | 1 | 6.56 | 17 | 88.94 | 2.052 | 1 | Yes |
| N-hexadecanoic acid (CID 985) | 256.43 | 1 | 1 | 5.20 | 14 | 92.004 | 1.763 | 1 | Yes |
| Phytyl palmitate (CID 6437053) | 534.95 | 2 | 0 | 11.75 | 28 | 88.58 | 1.813 | 2 | No |
| L-(+)-ascorbic acid 2,6-dihexadecanoate (CID 54722209) | 652.95 | 8 | 2 | 9.57 | 32 | 76.99 | 1.781 | 2 | No |
| Bis[di(trimethylsiloxy)phenylsiloxy] trimethylsiloxyphenylsiloxane (CID 6422911) | 793.52 | 7 | 0 | 5.17 | 17 | 91.55 | -0.192 | 1 | Yes |
| Z, z-6,27-hexatriactontadien-2-one (CID 5364674) | 516.94 | 1 | 0 | 12.00 | 31 | 86.43 | 2.211 | 2 | No |
| Hexadecanoic acid, 1-(hydroxymethyl)-1,2-ethanediyl ester (CID 99931) | 568.92 | 5 | 1 | 10.21 | 32 | 86.20 | 2.163 | 2 | No |
| Cyclotrisiloxane, 2,4,6-trimethyl-2,4,6-triphenyl- (CID 18901) | 408.68 | 3 | 0 | 3.55 | 3 | 100 | 0.586 | 0 | Yes |

Here, MW-molecular weight (g/mol); NHA- No. of hydrogen bond acceptor; NHD- No. of hydrogen bond donor; LogP-Predicted octanol/water partition coefficient; NRB- No. of rotatable bonds. IA-Intestinal absorption (% absorbed); TC-Total clearance (log ml/min/kg); NLV- Number of Lipinski’s Violation; DL- Drug Likeness

**Table S_2_:** The Protox 3.0 toxicity analysis of phytochemicals from EaNFL

| **Ligand name** | **BBB** | **LD50** | **HT**  **(Confidence)** | **AT** | **MTD** | **CT**  **(Confidence)** | **Toxicity Class** |
| --- | --- | --- | --- | --- | --- | --- | --- |
| (-) Epicatechin (CID 72276) | No | 2.428 | No (0.72) | No | 0.438 | No (0.84) | 6 |
| Caffeic acid (CID 689043) | No | 2.383 | No (0.57) | No | 1.145 | No (0.86) | 5 |
| Rutin hydrate (CID 5280805) | No | 2.489 | No (0.80) | No | 0.506 | No 0.64) | 5 |
| Rosmarinic acid (CID 5281792) | No | 2.811 | No (0.62) | No | 0.152 | No (0.90) | 5 |
| Myricetin (CID [5281672](https://pubchem.ncbi.nlm.nih.gov/compound/5281672)) | No | 2.497 | No (0.69) | No | 0.51 | No (0.99) | 3 |
| Kaempferol (CID [5280863](https://pubchem.ncbi.nlm.nih.gov/compound/5280863)) | No | 2.449 | No (0.68) | No | 0.531 | No (0.98) | 5 |
| Quercetin (CID 5280343) | No | 2.471 | No (0.69) | No | 0.499 | No (0.99) | 3 |
| trans-Cinnamic acid (CID 444539) | Yes | 2.103 | No (0.54) | No | 0.847 | No (0.83) | 5 |
| Guaiol (CID [227829](https://pubchem.ncbi.nlm.nih.gov/compound/227829)) | Yes | 1.789 | No (0.71) | No | 0.445 | No (0.67) | 5 |
| (-)-Aristolene (CID [530421](https://pubchem.ncbi.nlm.nih.gov/compound/530421)) | No | 1.565 | No (0.81) | No | -0.004 | No (0.68) | 5 |
| 2,3-dehydro-4-oxo-.beta.-ionone (CID 5363867) | Yes | 1.962 | No (0.65) | No | 0.471 | Yes (0.78) | 3 |
| Neophytadiene (CID [10446](https://pubchem.ncbi.nlm.nih.gov/compound/10446)) | No | 1.473 | No (0.79) | No | 0.272 | No (0.81) | 6 |
| Phytyl tetradecanoate (CID [14486554](https://pubchem.ncbi.nlm.nih.gov/compound/14486554)) | No | 1.871 | No (0.73) | No | -0.049 | No (0.78) | 6 |
| 1,5-diphenyl-2h-1,2,4-triazoline-3-thione (CID [2802516](https://pubchem.ncbi.nlm.nih.gov/compound/2802516)) | Yes | 2.81 | No (0.64) | No | 0.926 | No (0.87) | 4 |
| 13-docosen-1-ol, (z)- (CID [5354168](https://pubchem.ncbi.nlm.nih.gov/compound/5354168)) | No | 1.721 | No (0.89) | No | -0.34 | No (0.82) | 6 |
| Eicosen-1-ol, cis-9- (CID [5364523](https://pubchem.ncbi.nlm.nih.gov/compound/5364523)) | No | 1.657 | No (0.89) | No | -0.249 | No (0.82) | 6 |
| N-hexadecanoic acid (CID 985) | Yes | 1.44 | No (0.52) | No | -0.708 | No (0.74) | 4 |
| Phytyl palmitate (CID 6437053) | No | 1.933 | No (0.73) | No | -0.097 | No (0.78) | 6 |
| L-(+)-ascorbic acid 2,6-dihexadecanoate (CID 54722209) | No | 2.411 | No (0.95) | No | 0.336 | No (0.57) | 6 |
| Bis[di(trimethylsiloxy) phenylsiloxy] trimethylsiloxyphenylsiloxane (CID 6422911) | No | 2.749 | No (0.80) | No | 0.586 | No (0.75) | 5 |
| Z,z-6,27-hexatriactontadien-2-one (CID 5364674) | No | 2.03 | No (0.71) | No | -0.139 | No (0.72) | 6 |
| Hexadecanoic acid, 1-(hydroxymethyl)-1,2-ethanediyl ester (CID 99931) | No | 3.146 | No (0.88) | No | 0.069 | No (0.85) | 5 |
| Cyclotrisiloxane, 2,4,6-trimethyl-2,4,6-triphenyl- (CID 18901) | Yes | 2.129 | Yes (0.74) | Yes | 0.576 | No (0.78) | 5 |

Here, BBB- Blood Brain Barrier; LD50- Oral rat acute toxicity (mg/kg); HT- Hepatotoxicity; AT- AMES toxicity; MTD- Maximum tolerated dose for human (log mg/kg/day); CT- Cytotoxicity.

**Table S_3_:** MD score (Binding affinity) with the protein rcsb ID 5I6X

| **Protein ID** | **PubChem ID** | **Chemical Name** | **BA** |
| --- | --- | --- | --- |
| 5i6x | 5280805 | Rutin | -10.2 |
| 5i6x | 5281672 | Myricetin | -9.7 |
| 5i6x | 5280343 | Quercetin | -8.3 |
| 5i6x | 5280863 | Kaempferol | -8.2 |
| 5i6x | 72276 | (-)-Epicatechin | -8 |
| 5i6x | 5281792 | Rosmarinic Acid | -7.8 |
| 5i6x | 530421 | Aristolene | -7.6 |
| 5i6x | 3696 | Imipramine | -7.5 |
| 5i6x | 2802516 | 1,5-Diphenyl-1H-1,2,4-Triazole-3(2H)-Thione | -7.4 |
| 5i6x | 227829 | Guaiol | -7.3 |
| 5i6x | 6437053 | 53950-58-6 | -7.3 |
| 5i6x | 5363867 | 2,3-Dehydro-4-Oxo-.Beta.-Ionone | -6.8 |
| 5i6x | 444539 | Cinnamic Acid | -6.7 |
| 5i6x | 689043 | Caffeic Acid | -6.7 |
| 5i6x | 54722209 | 4218-81-9 | -6.7 |
| 5i6x | 10446 | Neophytadiene | -6.1 |
| 5i6x | 985 | Palmitic Acid | -5.9 |
| 5i6x | 5354168 | Cis-13-Docosenol | -5.7 |
| 5i6x | 5364523 | Gadoleyl Alcohol | -5.7 |
| 5i6x | 99931 | 1,2-Dipalmitoyl-Rac-Glycerol | -5.6 |
| 5i6x | 5364674 | Z,Z-6,27-Hexatriactontadien-2-One | -5.6 |

Here, BA = Binding Affinity (kcal/mol); RMSD- Root mean square deviation.

**Table S_4_:** MD score with the protein rcsb ID 4UJJ

| **Protein ID** | **PubChem ID** | **Chemical Name** | **BA** |
| --- | --- | --- | --- |
| 4uuj | 5280805 | Rutin | -9.4 |
| 4uuj | 5281672 | Myricetin | -8 |
| 4uuj | 5280343 | Quercetin | -7.7 |
| 4uuj | 5281792 | Rosmarinic Acid | -7.7 |
| 4uuj | 5280863 | Kaempferol | -7.6 |
| 4uuj | 72276 | (-)-Epicatechin | -7.5 |
| 4uuj | 72276 | (-)-Epicatechin | -7.5 |
| 4uuj | 530421 | Aristolene | -6.8 |
| 4uuj | 227829 | Guaiol | -6.6 |
| 4uuj | 227829 | Guaiol | -6.3 |
| 4uuj | 54722209 | 4218-81-9 | -6.1 |
| 4uuj | 2802516 | 1,5-Diphenyl-1H-1,2,4-Triazole-3(2H)-Thione | -6 |
| 4uuj | 689043 | Caffeic Acid | -5.8 |
| 4uuj | 5363867 | 2,3-Dehydro-4-Oxo-.Beta.-Ionone | -5.8 |
| 4uuj | 444539 | Cinnamic Acid | -5.7 |
| 4uuj | 99931 | 1,2-Dipalmitoyl-Rac-Glycerol | -5.4 |
| 4uuj | 5354168 | Cis-13-Docosenol | -5.2 |
| 4uuj | 6437053 | 53950-58-6 | -4.9 |
| 4uuj | 985 | Palmitic Acid | -4.5 |
| 4uuj | 10446 | Neophytadiene | -4.4 |
| 4uuj | 5364674 | Z,Z-6,27-Hexatriactontadien-2-One | -4.4 |
| 4uuj | 985 | Palmitic Acid | -4.3 |
| 4uuj | 5364523 | Gadoleyl Alcohol | -4.3 |

Here, BA = Binding Affinity (kcal/mol); RMSD- Root mean square deviation.

**Table S_5_:** MD score with the protein rcsb ID 6X3X

| **Protein ID** | **PubChem ID** | **Chemical Name** | **BA** |
| --- | --- | --- | --- |
| 6x3x | 5280805 | Rutin | -8.8 |
| 6x3x | 5281672 | Myricetin | -8.8 |
| 6x3x | 444539 | Cinnamic Acid | -8.6 |
| 6x3x | 72276 | (-)-Epicatechin | -8.1 |
| 6x3x | 5280343 | Quercetin | -8.1 |
| 6x3x | 2802516 | 1,5-Diphenyl-1H-1,2,4-Triazole-3(2H)-Thione | -7.9 |
| 6x3x | 3016 | Diazepam | -7.8 |
| 6x3x | 227829 | Guaiol | -7.7 |
| 6x3x | 5280863 | Kaempferol | -7.6 |
| 6x3x | 5281792 | Rosmarinic Acid | -7.5 |
| 6x3x | 689043 | Caffeic Acid | -7.3 |
| 6x3x | 54722209 | 4218-81-9 | -7 |
| 6x3x | 530421 | Aristolene | -6.9 |
| 6x3x | 5363867 | 2,3-Dehydro-4-Oxo-.Beta.-Ionone | -5.9 |
| 6x3x | 10446 | Neophytadiene | -5.5 |
| 6x3x | 6437053 | 53950-58-6 | -5.4 |
| 6x3x | 985 | Palmitic Acid | -5.2 |
| 6x3x | 5354168 | Cis-13-Docosenol | -5.1 |
| 6x3x | 99931 | 1,2-Dipalmitoyl-Rac-Glycerol | -4.8 |
| 6x3x | 5364523 | Gadoleyl Alcohol | -4.5 |
| 6x3x | 5364674 | Z,Z-6,27-Hexatriactontadien-2-One | -4.2 |

Here, BA = Binding Affinity (kcal/mol); RMSD- Root mean square deviation.
